# Genome Wide Association Identifies Novel Loci Involved in Fungal Communication

**DOI:** 10.1371/journal.pgen.1003669

**Published:** 2013-08-01

**Authors:** Javier Palma-Guerrero, Charles R. Hall, David Kowbel, Juliet Welch, John W. Taylor, Rachel B. Brem, N. Louise Glass

**Affiliations:** 1 Department of Plant and Microbial Biology, University of California, Berkeley, California, United States of America; 2 Department of Molecular and Cell Biology, University of California, Berkeley, California, United States of America; University of Washington, United States of America

## Abstract

Understanding how genomes encode complex cellular and organismal behaviors has become the outstanding challenge of modern genetics. Unlike classical screening methods, analysis of genetic variation that occurs naturally in wild populations can enable rapid, genome-scale mapping of genotype to phenotype with a medium-throughput experimental design. Here we describe the results of the first genome-wide association study (GWAS) used to identify novel loci underlying trait variation in a microbial eukaryote, harnessing wild isolates of the filamentous fungus *Neurospora crassa*. We genotyped each of a population of wild Louisiana strains at 1 million genetic loci genome-wide, and we used these genotypes to map genetic determinants of microbial communication. In *N. crassa*, germinated asexual spores (germlings) sense the presence of other germlings, grow toward them in a coordinated fashion, and fuse. We evaluated germlings of each strain for their ability to chemically sense, chemotropically seek, and undergo cell fusion, and we subjected these trait measurements to GWAS. This analysis identified one gene, NCU04379 (*cse-1*, encoding a homolog of a neuronal calcium sensor), at which inheritance was strongly associated with the efficiency of germling communication. Deletion of *cse-1* significantly impaired germling communication and fusion, and two genes encoding predicted interaction partners of CSE1 were also required for the communication trait. Additionally, mining our association results for signaling and secretion genes with a potential role in germling communication, we validated six more previously unknown molecular players, including a secreted protease and two other genes whose deletion conferred a novel phenotype of increased communication and multi-germling fusion. Our results establish protein secretion as a linchpin of germling communication in *N. crassa* and shed light on the regulation of communication molecules in this fungus. Our study demonstrates the power of population-genetic analyses for the rapid identification of genes contributing to complex traits in microbial species.

## Author Summary

Many phenotypes of interest are controlled by multiple loci, and in biological systems identifying determinants of such complex traits is challenging. Here, we genotyped 112 wild isolates of *Neurospora crassa* and used this resource to identify genes that mediate a fundamental but poorly-understood attribute of this filamentous fungus: the ability of germinating spores to sense each other at a distance, extend projections toward one another, and fuse. Inheritance at a secretion gene, *cse-1*, was associated strongly with germling communication across wild strains; this association was validated in experiments showing reduced communication in a *cse-1* deletion strain. By testing interacting partners of CSE1, and by assessing additional secretion and signaling factors whose inheritance associated more modestly with germling communication in wild strains, we identified eight other novel determinants of this phenotype. Our population of genotyped wild isolates provides a flexible and powerful community resource for the rapid identification of any varying, complex phenotype in *N. crassa*. The success of our approach, which used a phenotyping scheme far more tractable than would be required in a screen of the entire *N. crassa* gene deletion collection, serves as a proof of concept for association studies of wild populations for any organism.

## Introduction

In most filamentous ascomycete species, hyphae form an interconnected network or syncytium of multi-nucleate cells known as a mycelium [1]. In nature, the formation of a mycelium often occurs via the germination of wind-dispersed asexual spores (conidia) [2]. Upon landing on a suitable substrate, conidia germinate to form germlings that are capable of fusion via specialized structures called conidial anastomosis tubes (CATs) to form the interconnected mycelial network common in this group of organisms [3], [4]. The formation of mycelial networks by germling fusion increases cytoplasmic flow and is important for the distribution of nutrients, signals and organelles within the colony [5], [6].

Similar to cell fusion in other organisms, the process of germling fusion in the filamentous ascomycete fungus *Neurospora crassa* requires cell recognition and attraction, adhesion, cell wall remodeling and membrane merger [7]. Genetically identical germlings of *N. crassa* exhibit remarkable chemotropism to each other, which enhances the formation of the inter-connected hyphal network [8], [9]. A number of mutants have been identified in *N. crassa* that fail to undergo germling and hyphal fusion, including *nrc-1*, *mek-2* and *mak-2*, which are components of a conserved MAP kinase pathway [3], [10], [11], [12]. Other mutants of unknown biochemical function, such as *soft (so)*, also show defects in chemosensing and cell fusion [13], [14]. The components of the MAP kinase pathway (NRC1, MEK2 and MAK2) and SO are recruited in a rapid and oscillatory manner to the plasma membranes of germling pairs undergoing chemotropic interactions [12], [14]. The oscillation of MAK2 and SO to CAT tips has been proposed to allow genetically identical cells to alternate between two different physiological states associated with signal delivery or response [14], [15], [16]. Given the complex physiology of cell communication and fusion, many other genes and proteins likely play a role in this process.

*N. crassa* is a heterothallic, obligate outbreeding species that has been a model for the study of population structure and genetic variability of fungi in the wild [17], [18], [19], [20], [21]. Recent advances in nucleic acid sequencing technologies have allowed for large-scale sampling of wild populations in this model microbe, and we recently harnessed this strategy in a population structure analysis of *N. crassa* by RNA-seq [21]. Data from such a sequencing survey provides a dense map of genetic variants across the genome and raises the possibility of genome-wide association studies in *N. crassa*. Association mapping is a powerful tool to identify candidate cases in which genetic variation at the DNA level underlies differences between wild individuals in a trait of interest. This approach is in common use in human genetics but has had little application to date in model organism systems, although recent work has established the power of association studies in mapping the genetic basis of trait variation across wild individuals in *Drosophila* [22], [23], [24], *Arabidopsis* [25], [26], [27], [28] and sunflower [29]. In fungi [30], [31] and in most other organisms beside humans, studies seeking to use natural variation as a screening tool to map genotype to phenotype have been largely limited to experimental cross designs, which survey polymorphisms in only a few wild individuals.

Here we describe the results of the first genome-wide association analysis used to identify novel loci underlying trait variation in a microbial eukaryote. We applied an association strategy using wild isolates of *N. crassa* to identify the genetic basis of the complex trait of germling communication. Developing a detailed, quantitative assay well-suited to the medium-throughput association-mapping paradigm, we surveyed germling communication across wild *N. crassa* strains and mapped differences in this trait to DNA sequence variants. We subsequently tested the function of genes mapped in our association study by assessing the germling communication phenotype of deletion strains, revealing mutants that showed both decreased and increased germling fusion frequency. We also tested the effect of some gene deletions on MAK2 and SO oscillation during chemotropic interactions. And we localized within hyphae the protein product of the gene that showed the most significant association with germling communication phenotype, a homolog of mammalian neuronal calcium sensor-1 (NCS-1).

## Results

### Efficiency of germling communication varies across individuals in a wild population of *N. crassa*

Our previous study of the relatedness of wild *N. crassa* isolates from the Western hemisphere by RNA-seq revealed a well-defined population of 20 individuals from Louisiana [21]. To establish a larger set of genotyped Louisiana strains suitable for use in association mapping, we transcriptionally profiled an additional 92 Louisiana strains (Table S1). Analysis of the regulatory variation across the Louisiana population detected in these data will be reported elsewhere; here we used the RNA-seq reads to identify 1.09 million single-nucleotide polymorphisms (SNPs) in coding regions of the seven *N. crassa* chromosomes (Dataset S1). Phylogenetic analysis of these SNPs (Figure S1) indicated a set of 100 strains with little population substructure, including the smaller sample of Louisiana isolates that we had previously characterized [21]. We identified 81,614 SNPs at which the minor allele was present in >25% of strains, and which were equally distributed throughout the euchromatic regions of all seven chromosomes of *N. crassa* (Figure S2 and Dataset S2). Across the 9,730 protein-coding genes of the *N. crassa* genome (http://www.broadinstitute.org/annotation/genome/neurospora/MultiHome.html), the average gene harbored ∼10 high-frequency SNPs.

**Figure 1:**
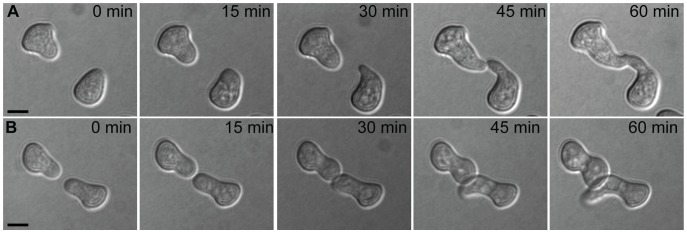
Chemotropic interactions and cell fusion between genetically identical conidial germlings. Each row shows one pair of germlings of the *N. crassa* laboratory strain FGSC 2489 during a fusion time-course. (A) Chemotropic interactions resulting in cell fusion. (B) Incomplete communication and fusion failure. Scale bar = 5 µm.

**Figure 2:**
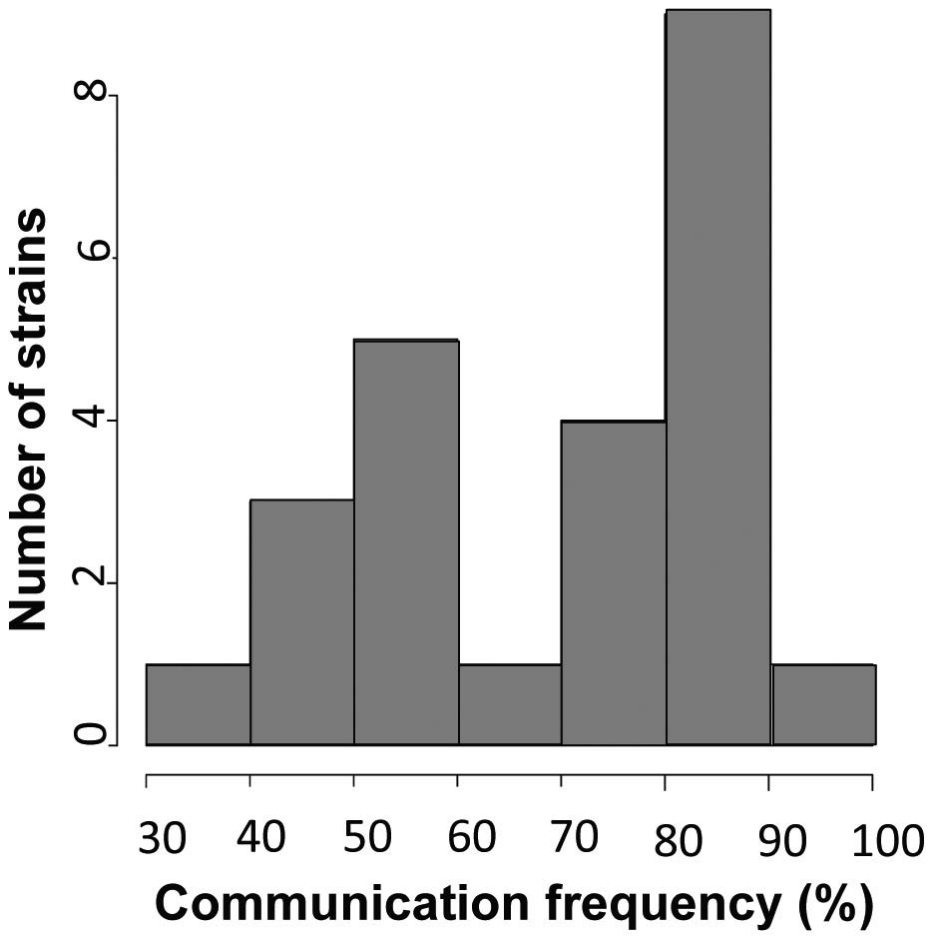
Germling communication frequency varies among wild *N. crassa* isolates from a single population in Louisiana. The *x*-axis shows the frequency of communication observed between genetically identical germling pairs for a given strain, and the *y*-axis reports the number of strains, among 24 Louisiana isolates, that exhibited the communication frequency denoted on the *x* axis.

To use our genotyped Louisiana strains to dissect the genetics of germling communication, we first developed a communication assay as follows. When genetically identical macroconidia of the *N. crassa* laboratory strain FGSC 2489 germinate near each other, ∼89% of the germlings within 15 µm of other germlings sense their neighbors, reorient their growth, and engage in cell fusion via CATs [3] (Figure 1A). The remaining germlings ignore each other, do not show chemotropism, do not form CATs and do not fuse (Figure 1B). We thus quantified communication by isolating macroconidia from each given wild strain, plating them on agarose minimal medium, and tabulating the percent of germling pairs exhibiting redirected CAT growth (communication) or fusion after 3–4 hours of incubation. Applying this procedure to 24 Louisiana strains showed that the germling communication trait varied among the wild isolates, from a high of 90% communication/cell fusion efficiency to a low of less than 40% communication (Table S2 and Figure 2).

### A Golgi-localized homolog of *neuronal calcium sensor*-1 is required for germling communication

**Figure 3:**
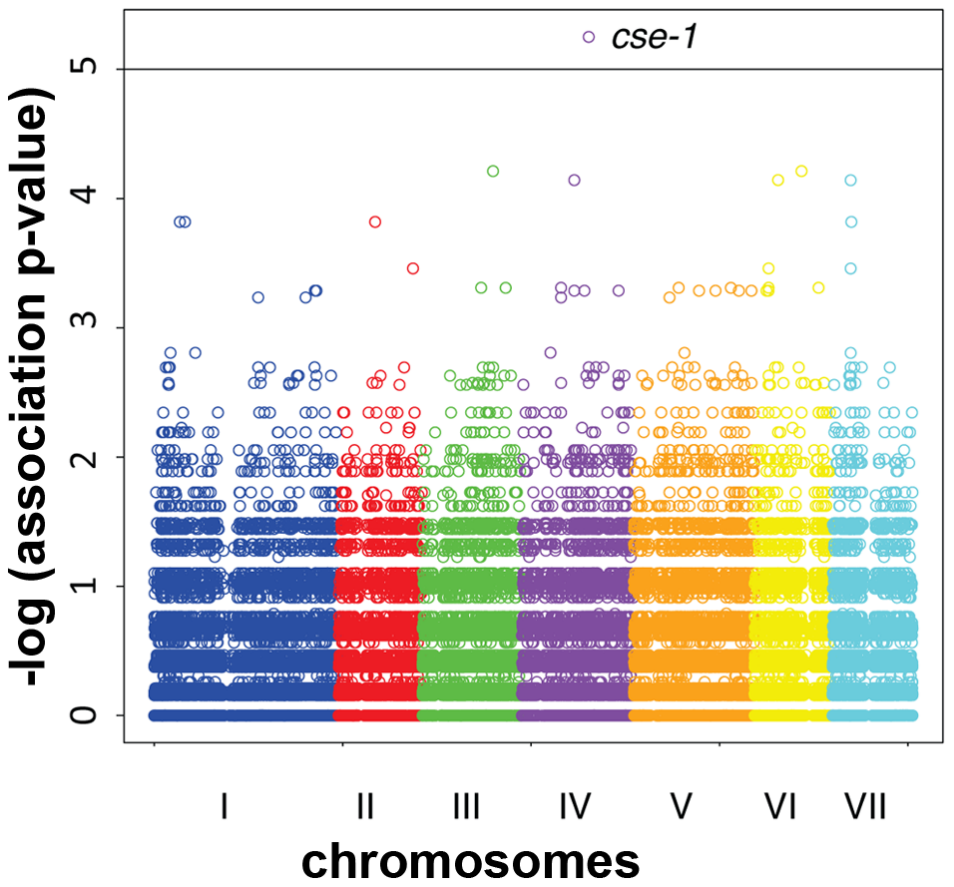
Genome-wide association of germling communication frequency on the seven chromosomes of *N. crassa*. Each data point represents one single-nucleotide polymorphism (SNP) at which inheritance was tested for association with frequency of germling communication across 24 wild strains. The *x*-axis reports SNP position on each chromosome as labeled with Roman numerals, and the *y*-axis reports the negative logarithm of the nominal association *p*-value by Fisher's exact test. The horizontal line indicates the significance threshold at which we would expect 0.1 SNP to score by chance. Association signals from three SNPs in *cse-1*, whose chromosomal positions are not distinguishable on this scale (see Figure 4), are visible as a single point as indicated; in each case the association *p*-value is 5.6×10^−6^, where 0.011 SNP is expected by chance.

**Figure 4:**
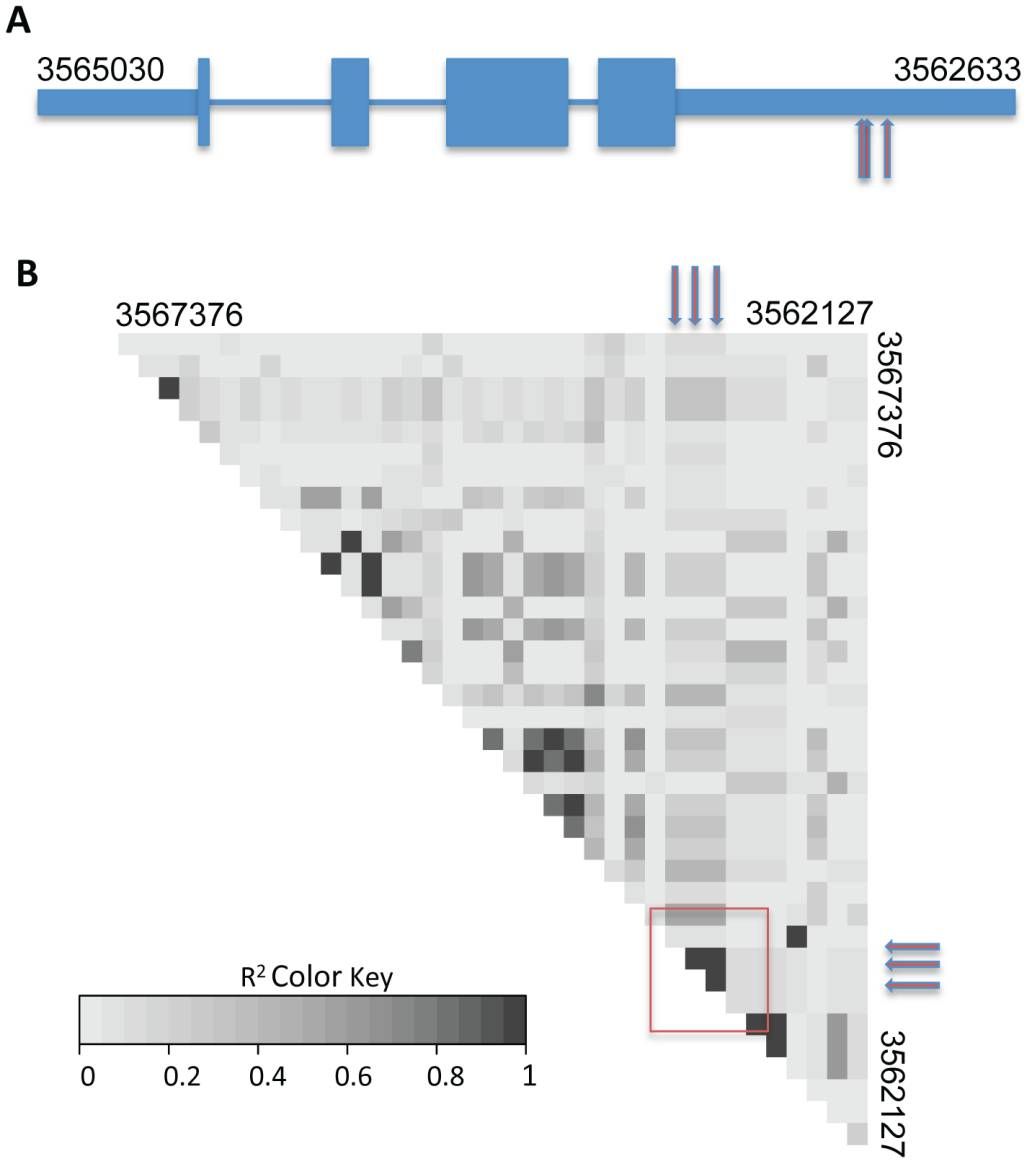
Variants in the 3′ UTR of *cse-1* (NCU04379) are associated with germling communication frequency. The remarkable precision of GWAS in the wild, Louisiana, *N. crassa* population is shown by identification of associated SNPs in just one region of one gene. (A) Cartoon of exon structure of CSE1. Narrow rectangles represent untranslated regions, wide rectangles represent exons and lines indicate introns. Red arrows represent positions of single-nucleotide polymorphisms at which inheritance associates strongly with germling communication in wild strains (SNP positions 3562999, 3562998, and 3562957 on chromosome IV). (B) Each grey box reports linkage disequilibrium (the degree to which one allele at one SNP preferentially appears in the population with one allele at a second SNP) as measured by r^2^ between one pair of high-frequency SNPs in a region of chromosome IV spanning *cse-1*. Red arrows represent the associating SNPs from (A), and the region of the heatmap reporting linkage disequilibrium between them is denoted with a red box.

To map loci underlying the variation in communication efficiency/cell fusion across our wild population, we first scored patterns of germling interactions as a qualitative, binary trait, such that the phenotype of a given individual was classified as either avidly or poorly communicating. We then used our set of genotypes at high-frequency SNPs to test each locus in turn for co-inheritance with the communication trait across the strains of the population, using a permutation strategy, described in Methods, to correct for multiple testing. This mapping calculation yielded 3 SNPs showing significant association with germling communication at a threshold at which we expected ∼0.01 SNP by chance (Figure 3 and Dataset S3). All three SNPs lay in the 3′ UTR of the gene NCU04379 with linkage disequilibrium decaying sharply around this peak (Figure 4); we detected no differential expression of NCU04379 between strains with avid germling communication and those whose germlings communicated poorly (data not shown).

**Figure 5:**
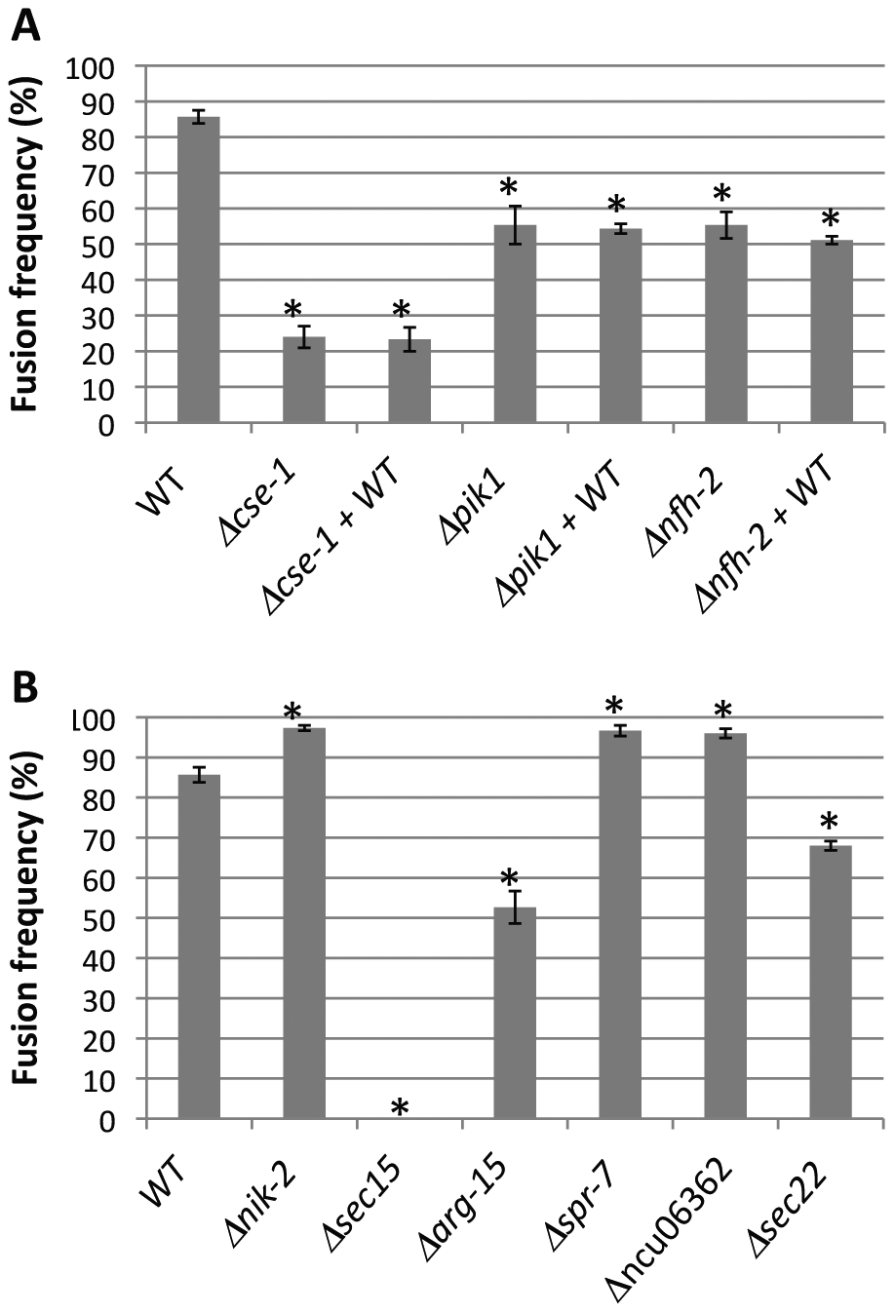
Nine novel genetic determinants of germling communication and fusion frequency. Each bar labeled with a single strain name reports the fusion frequency between germlings of wild-type FGSC 2489 (WT), or in a FGSC 2489 derivative harboring a deletion (Δ) of the indicated gene. Each bar labeled with a gene name+WT reports the fusion frequency observed when germlings of wild-type FGSC 2489 were mixed with germlings of the indicated deletion. (A) Germling communication by strains bearing deletions in the GWAS hit *cse-1* (see Figures 3 and 4) and its predicted interaction partners, *pik1* and *nfh-2*. (B) Germling communication in strains bearing deletions of genes ascertained using a permissive GWAS threshold (see Table 1). Asterisks indicate strains with communication significantly different from that of wild-type (Student's t-test, *p*<0.05). Bars indicate standard errors.

NCU04379 encodes CSE1, a homolog of the vertebrate neuronal calcium sensor-1 (NCS-1) and of Frq1p in *Saccharomyces cerevisiae* [32]. Deletion of *cse-1* in *N. crassa* results in a mutant that is viable, but sensitive to calcium stress and ultraviolet light, and which shows slightly impaired growth [33]. Similar to NCS-1 and Frq1p, CSE1 harbors a consensus signal for N-terminal myristoylation and four EF-hand domains (PF00036) predicted to be involved in calcium binding [32], [34], [35]. We hypothesized that CSE1 played a role in germling communication and that mutations in this gene would impact cell fusion behavior. Germling CAT fusion experiments validated this prediction, revealing a striking 3.6-fold reduction in the frequency of communication and cell fusion between Δ*cse-1* germlings relative to communication between germlings of the wild-type, isogenic strain from which the Δ*cse-1* strain was derived (Figure 5A). The defect was rescued by integration of a wild-type copy of *cse-1* at the *his-3* locus in the Δ*cse-1* strain, confirming the specificity of the phenotype to the *cse-1* lesion (Figure S3). To evaluate the ability of Δ*cse-1* germlings to respond to communication with wild-type isolates, we assayed Δ*cse-1* germlings positioned alongside those of the isogenic fusion-competent strain, and observed a defect similar to that of Δ*cse-1* germlings interacting with one another (Figure 5A). Thus, CSE1 is essential for chemotropic interactions, including the sensing of and response to the presence of a fusion-competent partner.

**Figure 6:**
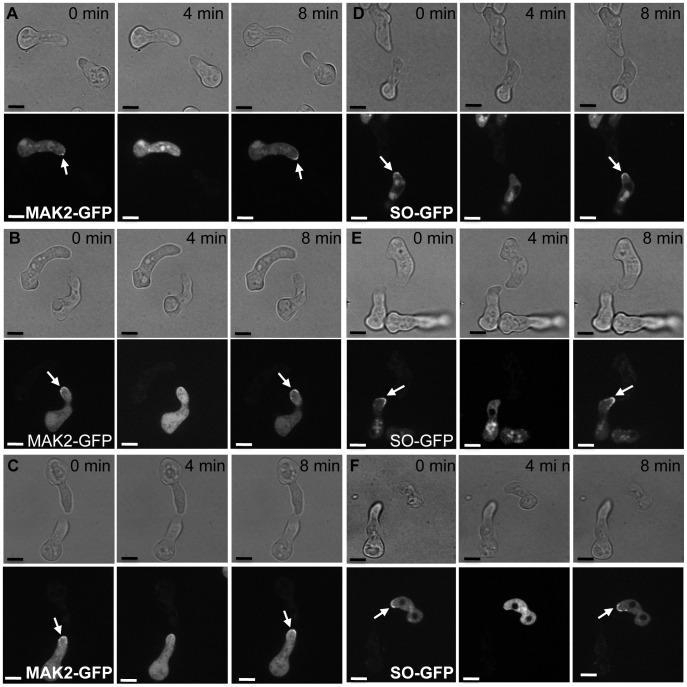
MAK2-GFP (A to C) and SO-GFP (D to F) oscillation in a wild type germling paired with Δ*cse-1* or Δ*nfh-2* germlings. Each panel shows frames from time-lapse visualization of one representative pair of germlings, in which one cell is a wild-type FGSC 2489 derivative expressing a communication protein tagged with GFP and the other cell is unmarked, of the FGSC 2489 background or a FGSC 2489 derivative harboring a single-gene deletion (Δ). Arrows show localization of MAK2 to CATs in panels A–C and SO localization to CATs in panels D–F. (A) Oscillation of MAK2-GFP in a wild-type germling (*his-3::Pccg1 mak-2-gfp*; *Δmak-2*) when paired with unmarked wild type germling. All germlings participating in chemotropic interactions showed oscillation of MAK2. (B) Oscillation of MAK2-GFP in a wild-type germling (*his-3::Pccg1 mak-2-gfp; Δmak-2*) when paired with an unmarked Δ*cse-1* mutant, in one of the rare instances in which chemotropic interactions were observed. (C) Oscillation of MAK2-GFP in a wild-type germling (*his-3::Pccg1 mak-2-gfp; Δmak-2*) when paired with a *nfh-2* germling in one of the few cases where chemotropic interactions were observed. (D) Oscillation of SO-GFP in a wild-type germling (*his-3::Pccg1 SO-gfp; ΔSO*) when paired with unmarked wild-type germling. Oscillation of SO was observed in all wild type-germlings participating in chemotropic interactions. (E) Oscillation of SO-GFP in a wild-type germling (*his-3::Pccg1 SO-gfp; ΔSO*) when paired with an unmarked Δ*cse-1* germling, in a case in which chemotropic interactions were observed. (F) Oscillation of SO-GFP in a wild-type germling (*his-3::Pccg1 SO-gfp; ΔSO*) when paired with unmarked Δ*nfh-2* germling, in one of the few cases in which chemotropic interactions were observed. Scale bar = 5 µm.

We next sought to learn if CSE1 acts before or after a required, chemotropic interaction event in germling fusion, the observable oscillations of MAK2 and SO to the tips of communicating CATs [14]. To address this question, we obtained a wild-type strain expressing either MAK2-GFP or SO-GFP, and we visualized the subcellular localization of the latter proteins during interactions between wild-type germlings and those of the Δ*cse-1* mutant background. In the few cases in which a Δ*cse-1* germling showed chemotropic interactions toward a wild-type germling, we observed normal recruitment and oscillation of both MAK2 and SO to wild-type germling tips (every ∼4 minutes) (Figure 6). In the ∼75% of cases in which a Δ*cse-1* germling and a wild-type germling showed no evidence of chemotropic interactions, MAK2 and SO did not localize or oscillate to CAT tips, but remained in the cytoplasm. We conclude that CSE1 acts upstream of the signaling that underlies chemotropic interactions, because in the rare instances where Δ*cse-1* germlings commit to chemotropic interactions and cell fusion, they successfully drove MAK2 and SO oscillation.

**Figure 7:**
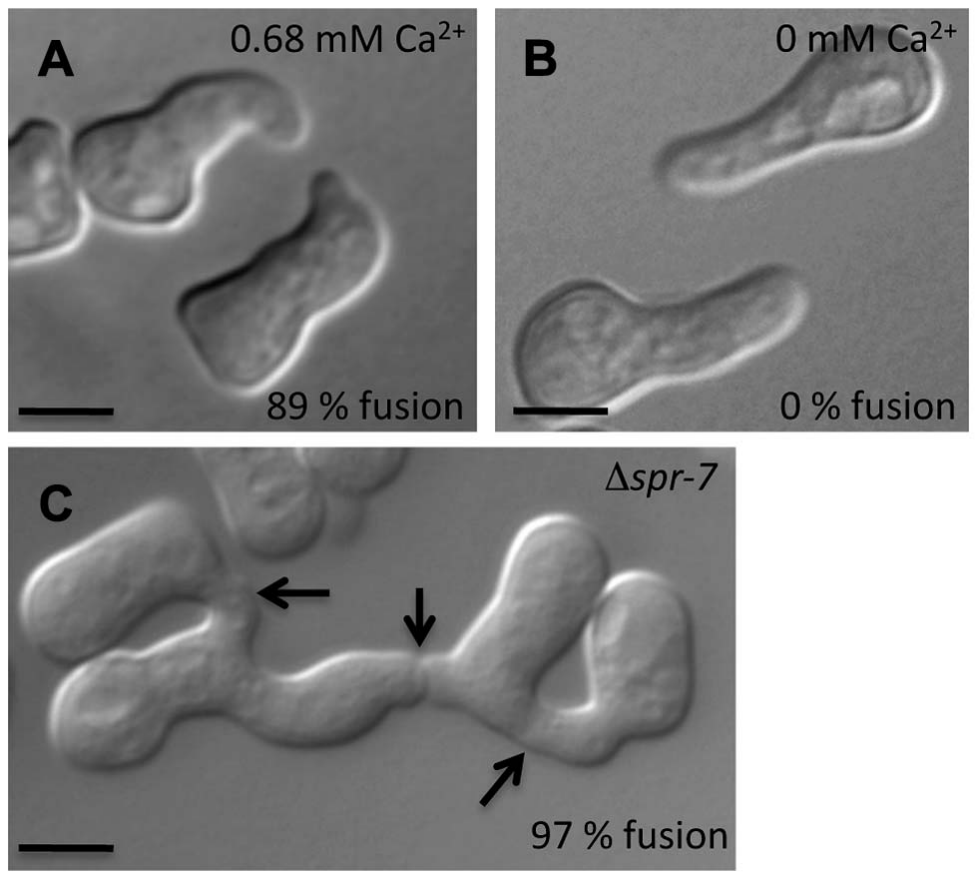
Conidial germling fusion phenotypes. (A) Representative micrograph of wild-type (FGSC 2489) germlings undergoing chemotropic interactions in standard laboratory medium (0.68 mM Ca^2+^) [71]. (B) Representative micrograph of failure of chemotropic interactions and cell fusion in wild type (FGSC 2489) germlings in standard laboratory media lacking Ca^2+^. Fusion frequencies from large-scale replicate analyses are in black text at the lower right of each image. (C) Germlings bearing a deletion in *spr-7* involved in multiple germling fusion events. Arrows indicate fusion points. Scale bar = 5 µm.

**Figure 8:**
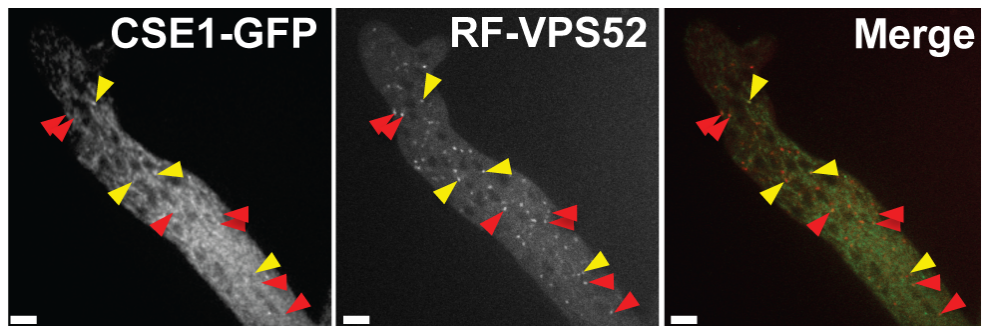
CSE1-GFP and RFP-VPS-52 colocalization. A heterokaryon between Δ*cse-1* (*cse-1-gfp::his-3*) *A*+*rfp-vps-52::his-3 A* showed co-localization of CSE1-GFP and the Golgi marker RFP-VPS-52 (yellow arrows). The red arrows indicate partial colocalization, likely due to cytoplasmic movement between frames. Scale bar = 5 µm.

The mammalian homolog of CSE1, NCS-1, functions during regulated exocytosis in response to calcium signaling [36], [37], and the yeast homolog Frq1p localizes to the Golgi membrane [38]. We reasoned that these attributes would likely be conserved in *N. crassa*. We first focused on the role of calcium; the Δ*cse-1* mutant shows growth sensitivity to excess calcium, as well as to calcium depletion [33]. We therefore hypothesized that calcium could be required for chemotropic interactions between *N. crassa* germlings, and to test this, we assayed fusion of wild-type germlings on growth medium depleted of Ca^2+^. The results (Figure 7B) bore out our prediction, with no detectable chemotropic interactions or CAT fusion in the absence of Ca^2+^. We next investigated the localization of CSE1 in *N. crassa*. For this purpose, we used a *Δcse-1* strain in which the *cse-1* allele with a C-terminal GFP tag had been integrated at the *his-3* locus. The introduction of the GFP-tagged *cse-1* allele restored wild-type growth and germling communication phenotype to the *Δcse-1* strain (Figure S3). We compared the localization of CSE1-GFP to that of the late Golgi marker VSP52 tagged with RFP [39], [40]. The results, shown in Figure 8, revealed colocalization of the CSE1 and VPS52, with CSE1-GFP also present in the cytoplasm.

### The Golgi secretion PI-4-kinase and a 14-3-3 regulator function in germling communication

Mammalian NCS-1 and *S. cerevisiae* Frq1p interact with phosphatidylinositol 4-kinase (Pik1p) [32], [37], a protein involved in secretion from the Golgi to the plasma membrane. As Frq1p is required for regulated exocytosis through Pik1p [38], we hypothesized that *N. crassa* homologs of components of this secretion pathway would play a role in germling communication. To test this hypothesis, we first assayed germlings carrying a deletion of the Pik1p homolog in *N. crassa*, NCU10397 (*pik1*), and observed a 1.5-fold reduction of germling communication (Figure 5A). A communication defect of similar magnitude was apparent when Δ*pik1* mutant germlings were assayed for interactions with wild-type fusion partners (Figure 5A). We next investigated 14-3-3 proteins, regulatory molecules that bind diverse signaling proteins [41] and in *S. cerevisiae* transport Pik1p from the nucleus to the cytoplasm [42]. Two members of this family have been identified in *N. crassa*, NCU03300 (*nfh-1*, encoding the DNA damage checkpoint component RAD24) and NCU02806 (*nfh-2*, encoding a 14-3-3 protein); we assayed germling communication in strains harboring deletions in each of these genes in turn. The results revealed no effect of the Δ*nfh-1* mutation (data not shown), but Δ*nfh-2* germlings communicated with one another at a frequency 1.5-fold less than that of isogenic wild-type germlings (Figure 5A), and Δ*nfh-2* conidia mixed with those of a wild-type strain exhibited a similar defect (Figure 5A). Echoing our findings from the Δ*cse-1* mutant, we observed normal oscillation of MAK2-GFP and SO-GFP to the CATs of wild-type germlings when they participated in chemotropic interactions with Δ*nfh-2* germlings, while wild-type germlings that did not communicate with those of the Δ*nfh-2* background showed uniquely cytoplasmic localization of MAK2-GFP and SO-GFP (Figure 6). Taken together, these data indicate that CSE1, PIK1, and NFH2 are each required for the calcium-dependent initiation of germling communication and chemotropic interactions, strongly suggesting their joint function in a Golgi secretion pathway involved in signaling to initiate germling fusion.

### Six additional secretion and signaling genes are involved in germling communication

Given the robust genetic association between *cse-1* genotype and germling communcation in wild strains (Figure 3), we reasoned that additional determinants of germling communication could be revealed by mining our genome-wide association data at lower significance levels. For this purpose, we re-examined our association results using a permissive threshold of *p*<0.015. Permutation testing estimated that 22% of loci reaching this level would be true positives (see methods); as such, independent gene-by-gene validation could uncover *bona fide* communication genes among this set, potentially both activators and repressors of the communication trait. We focused on genes annotated in secretion, kinase signalling pathways, or peptide hydrolysis in which SNPs showed association reaching our permissive significance cutoff. Of the 18 genes that fit this description and for which deletion strains were available and viable (Table 1), deletion of six genes had significant impact on communication frequencies as compared to a wild-type strain (Figure 5B). The most extreme phenotype, a complete failure of chemotropic interactions and CAT fusion, was observed in the deletion strain for the exocyst complex component *sec15* (NCU00117) (Table 1; Figure 5B). The latter mutant also exhibited slower growth, reduced conidiation, and slower conidial germination. Deletion of two additional genes, the protein transporter *sec22* (NCU06708) and the acetylornithine-glutamate transacetylase *arg-15* (NCU05622) [43], also compromised fusion frequency (68%±2 and 53%±4, respectively) (Figure 5B). Remarkably, deletion of each of three genes heightened germling communication and fusion frequencies (Figure 5B): a GTPase activating protein (NCU06362; 96%±2), the nonidentical kinase-2 *nik-2* (NCU01833; 97%±0.7), and the secreted subtilisin-like serine protease *spr-7* (NCU07159; 97±1.3). The elevated fusion frequency in each of these strains contrasts with any known germling fusion mutant, all of which reduce or eliminate chemotropic interactions or cell fusion, and highlights the ability of association mapping to pinpoint negative regulators as well as genes with a positive role in cell communication. In each mutant with heightened fusion frequency, germlings were also often involved in fusion events with more than one germling (multiple fusion events) (26.33%±5.24 in ΔNCU06362, 21.33%±1.8 in *Δnik-2*, and 20.66%±4.07 in *Δspr-7*; Figure 7C). By contrast, multiple germling fusion events was a phenotype only observed at a low level in a wild-type strain (2%±2).

**Table 1:** GWAS of germling communication in wild *N. crassa* isolates and validation in laboratory deletion strains.

Gene	GWAS *p*-value	Annotation	Communication % ± SE in deletion strains
NCU04379	5.6·10^−6^	Neuronal calcium sensor-1 (CSE1)	24±3.1*
NCU06362	7.2·10^−5^	GTPase activating protein	96±2*
NCU06708	5.2·10^−4^	Protein transporter *SEC22*	68±2*
NCU04252	2·10^−3^	SNARE docking complex subunit-Sec1	83±2.4
NCU03819	2.8·10^−3^	COPII coat assembly protein Sec16	85±3
NCU01833	3·10^−3^	Non identicak kinase 2 (NIK-2)	97±0.7*
NCU06240	3·10^−3^	protein kinase A catalytic subunit-1	87±0.7
NCU00196	3·10^−3^	rho GTPase activator	85±2.4
NCU00477	3·10^−3^	carboxypeptidase Y	83±0.7
NCU02338	6·10^−3^	secreted protein	89±0.7
NCU00665	8.8·10^−3^	Ser/Thr protein phosphatase	87±1.2
NCU04005	9·10^−3^	casein kinase-1b	87±2.4
NCU05808	9·10^−3^	serine/threonine protein kinase	89±1.3
NCU00117	0.01	exocyst complex component Sec15	0*
NCU03658	0.01	secretion pathway protein Sls2/Rcy1	88±1.2
NCU03659	0.011	serine/threonine-protein kinase	87±2.4
NCU05622	0.011	acetylornithine-glutamate transacetylase (ARG-15)	53±4*
NCU07159	0.011	secreted subtilisin-related serine protease (SPR-7)	97±1.3*
NCU06949	0.011	subtilisin-like proteinase Mp1- secreted	91±0.7

* indicates significant difference with respect to WT (Student's t-test, *p*<0.05).

To investigate further the novel gain-of-fusion phenotype, we focused on the putative secreted serine protease *spr-7*. We first confirmed that the introduction of an ectopic copy of *spr-7* at the *his-3* locus restored hyphal communication of the *spr-7* deletion strain to wild-type levels, establishing the deletion as the sole cause of the increased communication phenotype (Figure S3). We next asked whether the presence of wild-type germlings would be sufficient to complement the Δ*spr-7* phenotype during communication. Assays of Δ*spr-7* germlings mixed with those of a wild-type strain confirmed this hypothesis, revealing a fully wild-type communication phenotype (fusion frequency 82%±3), a striking contrast to the failure to communicate with wild-type germlings we had noted in Δ*cse-1, Δpik1* and *Δnfh-2* mutants (Figure 5 and see above). These results support a model in which secreted SPR-7 from wild-type germlings acts in a cell-non-autonomous fashion to restrict communication and CAT fusion between wild type germlings.

## Discussion

In *N. crassa*, genetically identical germlings chemotropically sense partner cells and undergo mutual recognition-directed growth and cell fusion [14], [15], [16]. The molecular basis of this phenotype is only partly understood, and tools to identify candidate genes involved in fusion are at a premium in the field. In this work, we genotyped more than 100 wild *N. crassa* isolates, advanced our understanding of germling communication and fusion, and established this population as a powerful resource for high-resolution association mapping that can be used with any variable phenotype. Our study is the first to illustrate the utility of genome-wide association mapping to identify novel loci underlying trait variation in a microbe. We anticipate that this methodology will be a powerful and generally applicable tool in future genetic study of many eukaryotic microbes, owing to the small genome sizes and deeply-sampled populations of a number of species, particularly filamentous fungi.

**Figure 9:**
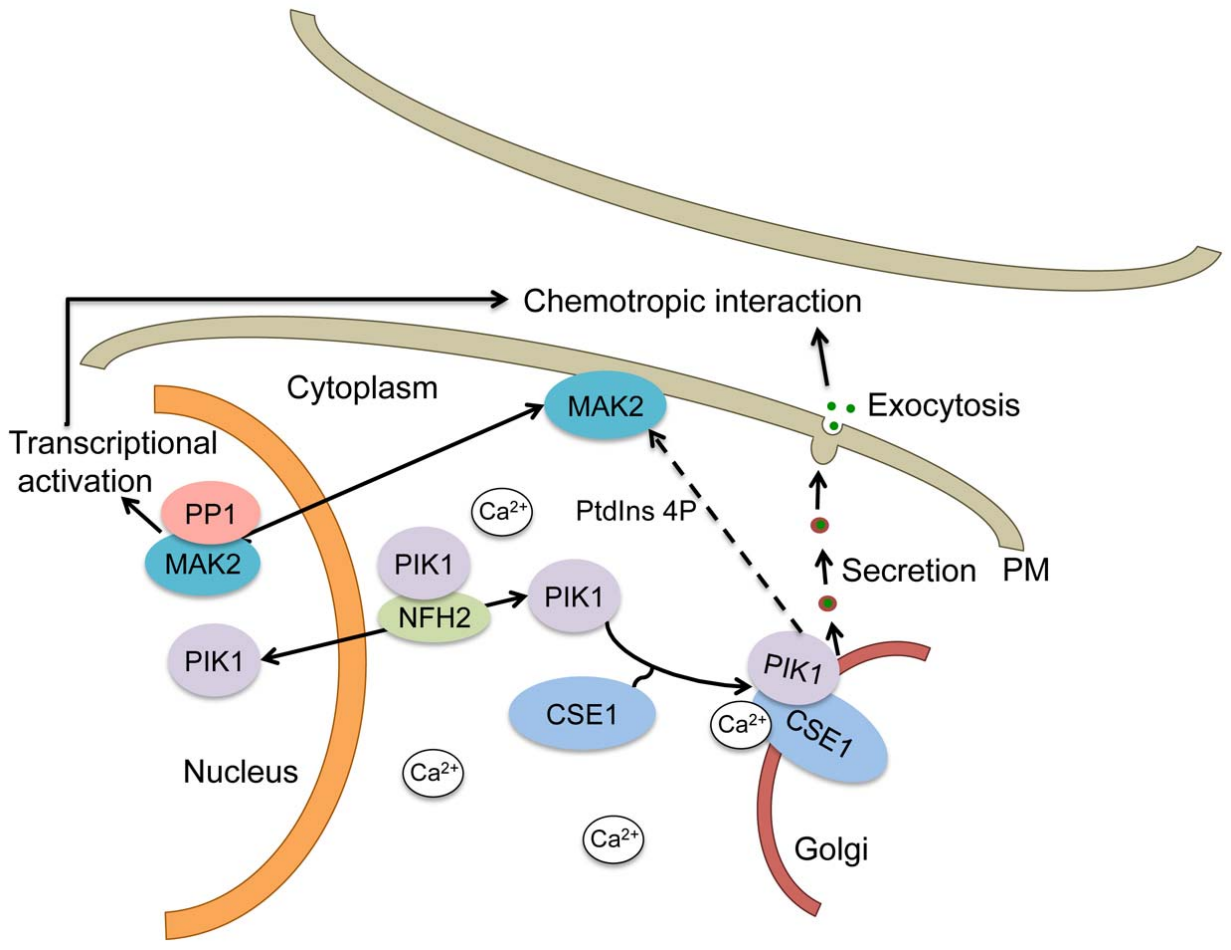
Schematic of a mechanistic model for the regulation of secretion by CSE1, PIK1 and NFH2 during germling chemotropic interactions and cell fusion. PIK1 interaction with CSE1 allows its recruitment to Golgi membranes, where it plays a role in PtdIns4p production and the regulated secretion of the signals needed for chemotropic interactions. In addition, PtdIns4p may play a role in MAK2 activation, which is required for chemotropic interactions and cell fusion. PIK1 is predicted to shuttle between the cytosol and the nucleus in a manner that is dependent on the BMH1 homolog NFH2.

The top hit from our association analysis was *cse-1*, which is homologous to a neuronal calcium sensor gene in animals that shows nervous-system-specific expression and neuron-specific phenotypes; neurons, like hyphae in filamentous fungi, are a highly polarized tissue. Neuronal calcium sensor-1 (Frequenin) is a myristolylated protein with four EF hands that functions as a calcium ion sensor for modulation of syntaptic activity and secretion [34], [44], [45], [46]. Our analysis revealed a near-complete loss of cellular communication during germling fusion in a *N. crassa* Δ*cse-1* mutant. In animals and in *S. cerevisiae*, NCS-1/Frq1p and Bmh1p-Bmh2p regulate phosphatidylinositol 4-kinase/Pik1p, with Bmh1p-Bmh2p mediating the nucleocytoplasmic shuttling of Pik1p [42]. NCS-1/Frq1p promotes association of Pik1p with the Golgi membrane, which is required for its role in regulated exocytosis [37], [38]. Our results established that in *N. crassa*, CSE1 localized to the Golgi and that deletion of *pik1* or *nfh-2* phenocopied a *cse-1* deletion strain. These observations together support a model in which, in *N. crassa*, CSE1, PIK1 and NFH2 regulate exocytosis of an unidentified ligand and/or receptor, perhaps initiated via calcium signaling, which is important for establishing communication between cells and subsequent chemotropic interactions (Figure 9). Recently, an essential kinase (MSS-4) involved in the generation of phosphatidylinositol 4,5-bisphosphate (PtdIns(4,5)P(2)) was found to localize to contact points between germlings during cell fusion [47], indicating that the generation of different phosphatidylinositol phosphate moieties may regulate different aspects of germling fusion.

A role for phosphorylation is suggested by our finding that the defect in germling communication observed in the *Δcse-1, Δpik1* and *Δnfh-2* mutants correlates with an absence of oscillation of MAK2 and SO to CAT tips, because MAK2 kinase activity has been shown to be required for chemotropic interactions and MAK2 and SO oscillation [14]. In *S. cerevisiae*, Pik1p is required for full activation of the MAP kinases Fus3p and Hog1p and repression of Kss1p [48], and the Fus3p ortholog in *N. crassa* is MAK2 [10]. It is therefore tempting to speculate that the activation of PIK1 by CSE1 may play an important role in germling communication by affecting activation of MAK2, thus modulating MAK2 phosphorylation targets as well as downstream transcriptional targets required for germling fusion (Figure 9).

In addition to our mapping of *cse-1* as a determinant of variation in germling communication across wild *N. crassa*, further mining of our association results led to the identification and validation of six other genes associated with CAT fusion. Of these, one gene, *sec15*, is a homolog of a component of the exocyst complex in *S. cerevisiae*, a multiprotein complex that localizes at the bud tip and is associated with exocytosis [49]. Our results indicated that *sec15* is essential for CAT fusion in *N. crassa*. Likewise, our results revealed a defect in germling communication and fusion frequency in a strain bearing a deletion in a homolog of *SEC22* in *N. crassa*, NCU06708; in *S. cerevisiae*, Sec22p assembles into a SNARE complex and plays a role in ER-Golgi protein trafficking [50]. Our demonstration that *cse-1*, *pik1*, *nfh-2*, *sec15*, and *sec22* are all required for germling communication establishes the importance of protein secretion and trafficking for chemotropic interactions and cell fusion in *N. crassa*.

Our results also established that mutation of the acetylornithine-glutamate transacetylase *arg-15* [43] confers a defect in germling communication. The homolog of *arg-15* in *S. cerevisiae*, Dug2p, is involved in degradation of the antioxidant glutathione and other peptides containing a gamma-glu-X. *dug2* mutants show deficient utilization of glutathione [51], which reacts non-enzymatically with reactive oxygen species and detoxifies oxidatively stressed cells [52]. A role for redox reactions in germling communication through *arg-15* would dovetail with reports that mutants in components of the NADPH oxidase complex, which is involved in redox signaling, are defective in CAT fusion [9].

Our work has uncovered a new category of fusion mutants that exhibited germling fusion frequencies higher than those of wild-type, and which displayed multiple fusion events. Of the genes whose deletions gave rise to this striking phenotype, one encoded an uncharacterized predicted GTPase activating protein (GAP) (NCU06362). NCU06362 contains a TBC domain (PF00566) and is a paralog of *GYP5* in *S. cerevisiae*; Gyp5p is involved in the recruitment to sites of polarized growth of the BAR domain protein Rvs167p, which has been implicated in exocytosis at the bud tip [53]. Rvs167p interacts with a second BAR domain protein, Rvs161p, and together this complex plays a role in receptor-mediated endocytosis [54]. Gyp5p also has *in vitro* GAP activity towards Ypt1p, which is involved in ER-to-Golgi trafficking, and towards Sec4p, which regulates exocytosis [55]. Thus, the increase in germling fusion frequencies observed in the ΔNCU06362 mutant could be due to alterations in secretion or in the reduction of endocytosis of a receptor involved in germling communication.

A second gene whose deletion enhanced hyphal communication, *spr-7*, encodes a secreted subtilisin-related serine protease, part of a family whose members carry out a wide range of peptidase activities [56]. The increase in fusion frequency and germlings involved in mutiple fusion events in the Δ*spr-7* mutant suggests that SPR-7 may be responsible for the degradation of a peptide required for extracellular communication (Figure 9). The nature of the extracellular ligand and receptor(s) that guide chemotropic interactions during cell fusion in *N. crassa* is currently unknown. In fungi, secreted peptides involved in extracellular communication have not been reported, apart from peptide pheromones involved in mating [57], [58] or small secreted proteins with antifungal properties [59], [60]. The genes we have uncovered here will serve as targets for future genetic and biochemical efforts to identify extracellular ligands and receptors involved in germling communication and cell fusion in *N. crassa*.

Our results also revealed an increase in germling communication in a *nik-2* deletion strain. This gene encodes a histidine kinase, a member of a canonical two-component signal transduction pathway and part of an 11-member family in *N. crassa*. No phenotype for the *Δnik-2* mutant has been previously reported [61]. However, other histidine kinases affect MAPK signal transduction pathways in fungi, including *nik-1*, a member of the osmoregulatory OS-2 pathway in *N. crassa* [62], and the histidine kinase Sln1p, which regulates the Hog1p MAPK pathway in *S. cerevisiae* [63]. We hypothesize that the increase in fusion frequencies in the absence of *nik-2* may stem from a defect in the regulation of the MAK2 phosphorylation pathway, leading to a hyper-activated state during chemotropic interaction (Figure 9). Further research will be necessary to elucidate the specific role of *nik-2* in this process.

By identifying multiple novel determinants of germling communication, our results underscore the power of association studies for the mapping of genes to phenotypes in wild populations. Importantly, our *N. crassa* population is particularly amenable to GWAS, with little discernable population structure and low linkage disequilibrium, allowing the detection of strong association to finely resolved loci. These attributes of *N. crassa* stand in contrast to *S. cerevisiae*, where GWA studies are hampered by a mosaic and heterogenous population structure [64]. Our relatively modest, medium-throughput phenotyping of a quantitative phenotype in wild individuals compares favorably with the high-throughput approach that would be required to survey the >9000 strains of the *N. crassa* deletion collection [65], not only by saving 98% of the labor, but in enabling analysis of all genes, including those that are essential. However, our molecular follow-up of GWAS hits was aided by the availability of a near-full genome deletion strain collection for *N. crassa*. When the central question, as in our work, is to infer novel function for poorly annotated genes, comparing a given gene's deletion strain and the isogenic wild-type strain is a straightforward and precise approach that obviates potential complications from epistasis in allele-swapping experiments. Our GWAS method also compares favorably to two-parent crossing schemes for the dissection of natural variation [66]: first, because linkage blocks in our outbreeding population often contain a single gene, whereas more than 50 can be contained in those resulting from just one cross [67], and second, because we sample phenotypes that vary among multiple individuals and not just those that differ between two parents. With the availability of our collection of 112 genotyped individuals to the fungal genetic community, future studies will require only phenotyping to map the molecular basis of trait variation using the strategy we have pioneered here. And as population-genomic resources are developed in many taxa, we anticipate that association mapping will be successfully applied in other species, within and outside the fungal kingdom.

## Methods

### Strains and growth conditions

All 112 strains used in this study were isolated from Louisiana, USA (Table S1) and are available from the Fungal Genetics Stock Center (FGSC) [68].

The deletion mutants used in these study were generated by the *Neurospora* Genome Project [65], [69] and are administered by the FGSC [70]. The *rfp-vps-52* transformant was generously provided by Barry Bowman [40]. All strains were grown on Vogel's medium [71] and all crosses were performed on Westergaard's synthetic cross medium [72]. The *his-3 A* mutant (FGSC# 6103) and a *his-3 a* strain (FGSC #9716) were used as females in crosses with deletion mutants. Progeny bearing the deletion mutations and the *his-3* mutation were isolated and used in complementation experiments.

### RNA isolation and cDNA synthesis

Total RNA was isolated for each of the 112 strains listed in Table S1. Strains were grown for 16 hrs on cellophane on Bird medium [73]. Mycelia were harvested and immediately added to 1 mL of TRIzol reagent (Invitrogen Life Technologies) [74] and zirconia/silica beads (0.2 g, 0.5-mm diameter; Biospec Products). Cells were disrupted using a MiniBeadBeater instrument (Biospec Products) at maximum speed for 30 seconds twice in succession. Total RNA was extracted according to the manufacturer's protocol for TRIzol (Invitrogen) and quantified on a Bioanalyzer (Agilent).

For polyA RNA purification, 10 µg of total RNA was bound to dynal oligo(dT) magnetic beads (Invitrogen 610.02) two times, using the manufacturer's instructions. Purified polyA RNA was fragmented by metal-ion catalysis [75] using fragmentation reagents from Ambion (AM12450). For first strand cDNA synthesis 1 µg fragmented polyA RNA was incubated with 3 µg random hexamers (Invitrogen 48190-011), and incubated at 65°C for 5 minutes and then transferred to ice. 1st strand buffer (Invitrogen 18064-014) was added to 1× final concentration (4 µL). Dithiothreitol (DTT), dNTPs and RNAseOUT (Invitrogen 10777-019) were added to 100 mM, 10 mM, and 20 U/20 µL respectively, and the sample was incubated at 25°C for 2 minutes. 200 U of Superscript II (Invitrogen 18064-014) were added and the sample was incubated at 25°C for 10 minutes, 42°C for 50 minutes and 70°C for 15 minutes.

For second strand synthesis, 51 µL of H_2_O, 20 µL of 5× second strand buffer (Invitrogen 10812-014), and dNTPs (10 mM) were added to the first strand cDNA synthesis mix and incubated on ice for 5 minutes. RNaseH (2 U) (Invitrogen 18021-014), DNA pol I (50 U) (Invitrogen 18010-017) were then added and the mixture was incubated at 16°C for 2.5 hours.

### Library construction and sequencing

End-repair was performed by adding 45 µL of H_2_O, T4 DNA ligase buffer with 10 mM ATP (NEB B0202S) (10 µL), dNTP mix (10 mM), T4 DNA polymerase (15 U) (NEB M0203L), Klenow DNA polymerase (5 U) (NEB M0210S), and T4 PNK (50 U) (NEB M0201L) to the sample and incubating at 20°C for 30 minutes. A single base was added each to cDNA fragment by adding Klenow buffer (NEB M0212L), dATP (1 mM), and Klenow 3′ to 5′ exo- (15 U) (NEB M0212L). The mixture was then incubated at 37°C in for 30 minutes.

Standard Illumina adapters (FC-102-1003) were ligated to the cDNA fragments using 2× DNA ligase buffer (Enzymatics L603-HC-L), 1 µL of adapters, and DNA ligase (5 U) (Enzymatics L603-HC-L). The sample was incubated at 25°C for 15 minutes. The sample was purified in a 2% low-melting point agarose gel, and a slice of gel containing 200-bp fragments was removed and the DNA purified. The polymerase chain reaction (PCR) was used to enrich the sequencing library. A 10-µL aliquot of purified cDNA library was amplified by PCR. PCR cycling conditions were: a denaturing step at 98°C for 30 seconds, 14 cycles of 98°C for 10 seconds, 65°C for 30 seconds, 68°C for 30 seconds, and a final extension at 68°C for 5 minutes. All libraries were sequenced using an Illumina Genome Analyzer-II using standard Illumina operating procedures. RNAseq data for all strains used in these analyses has been deposited in Gene Expression Omnibus (http://www.ncbi.nlm.nih.gov/geo/; accession no. GSE45406; GSM1103708-GSM1103819).

### SNP identification and phylogenetics

Mapping of RNA-seq reads to the genome sequence of *N. crassa* strain FGSC 2489 [76] and calling of single nucleotide polymorphisms (SNPs) was carried out with Maq [77]. All RNA-seq reads that mapped to multiple locations were eliminated from analysis, as were SNPs located in regions of low consensus read quality. These variants were further filtered to retain only those that were bi-allelic, yielding a complete data set of 1.09×10^6^ SNPs (Dataset S1) which were used as input into phylogenetic inference with FastTree; because patterns of inheritance in one strain, JW168, were suggestive of misclassification (data not shown) we did not include this strain in the tree shown in Figure S1. For markers used as input into calculations of genetic association with the germling communication phenotype (see below), we filtered the complete SNP set to retain only sites at which the minor allele was present at >25% frequency (Dataset S2).

### Conidial germling fusion frequency measurements

For germling communication assays, each strain was grown on Vogel's minimal media [71] in slant tubes for 4–6 days or until significant conidiation occurred. Conidial suspensions were prepared by collecting conidia with wood sticks and suspending in 600 µl of sterile distilled water. The conidial suspension was filtered by pouring over cheesecloth to remove hyphal fragments. Conidia were diluted to a concentration of 3×10^7^ conidia/ml and 300 µl of this final mixture were spread either on an agar or agarose minimal-medium plates. The plates were incubated for 3–4 hours at 30°. At each of 2–3 timepoints for each strain, agar squares of 1 cm were excised and observed with a Zeiss Axioskop 2 using a 403 Plan-Neofluor oil immersion objective. For image acquisition DIC images were taken with a Hamamatsu Orca 03 camera (Hamamatsu, Japan) using the iVision Mac4.5 software and a Zeiss Axioimager microscope. Fusion events were counted for 50 germling pairs in each of 2–3 biological replicates.

### Complementation analysis

Complementation experiments were done using the pMF272 plasmid system [78] to insert a wild type copy of the deleted gene into the intergenic region 3′ of the *his-3* locus; transformants were subsequently analyzed for germling fusion frequencies. Wild type copies of genes were amplified using Taq polymerase from New England Biolabs (Ipswich, CA, USA). Primers were designed to amplify the coding regions and also contained an added restriction enzyme site. The amplified DNA fragments were TOPO (Invitrogen) cloned, cut with restriction enzymes and ligated into restriction enzyme-digested pMF272 plasmid. The ligated DNA was used to transform *Escherichia coli* (DH5a), and the plasmid isolated from individual transformants. The DNA sequence of each plasmid was determined; plasmids containing wild type copies of the genes were used for complementation experiments.

### Confocal microscopy

Some mutants showing reduced fusion frequencies were further characterized by studying the ability of the mutant germlings to induce recruitment of MAK2-GFP or SO-GFP to the plasma membrane of opposing germlings as described by Fleißner *et al* [13]. Conidia from MAK2-GFP and SO-GFP strains were mixed with equal amounts of conidia from the respective deletion mutants and samples were prepared for microscopy as described above. Images were taken at two-minute intervals using a Leica SD6000 microscope with a 100×1.4 NA oil-immersion objective equipped with a Yokogawa CSU-X1 spinning disk head and a 488-nm laser controlled by Metamorph software (Molecular Devices, Sunnyvale, CA).

To visualize CSE1-GFP and RFP-VPS-52 localization, the strains were grown on Vogel's MM plates overnight and squares of 1 cm were excised and examined in the same confocal microscope explained above using the 488-nm laser for GFP and 563 nm laser for RFP. To study co-localization of both proteins, heterokaryons were made by mixing conidia from both strains in the center of a plate and incubating them overnight to allow cell fusion and cytoplasmic mixing from both strains. The samples were prepared and imaged as explained above.

### Whole-genome association mapping

We used germling communication phenotype measurements in biological triplicate from 24 Louisiana strains in a genome-wide association analysis as follows. For each strain, we first calculated the average communication frequency across all replicates and timepoints to yield a final quantitative communication measurement. We then converted the latter value to a qualitative score: we calculated the grand mean and standard deviation of communication frequency across all strains, and we classified a given strain as low-communicating if its communication measurement was more than one standard deviation below the grand mean, and high-communicating otherwise. We then tested each marker in turn, from our set of SNPs with >25% minor allele frequency (see above), for co-inheritance with this qualitative communication score using Fisher's exact test [79]. To evaluate the experiment-wise false discovery rate at a given Fisher's *p*-value threshold *p_thresh_*, we shuffled the vector of phenotype category values among strains, repeated the association test, and tabulated the number of SNPs with Fisher's *p*-value<*p_thresh_*, in this null data set. Averaging over 1000 such permutations yielded an expectation of 0.011 SNPs called at *p_thresh_* = 5.6×10^−6^ and 652 SNPs at *p_thresh_* = 0.015, under a null model of no true association. Given the 3 and 837 SNPs, respectively, reaching these levels in the real data (Dataset S3), false discovery rates at these thresholds were 0.4% and 78%, respectively. Linkage disequilibrium in Figure 4 was calculated between all high-frequency SNPs in the region of *cse-1* using the LDcorSV package in R.

## Supporting Information

Dataset S1 Single-nucleotide polymorphism (SNPs) in Louisiana strains of *N. crassa*. Each row reports inheritances at one genic SNP and each column reports inheritances in one Louisiana strain. Row names indicate SNP chromosome (supercontig) and position in release 10 of the *N. crassa* genome (http://www.broadinstitute.org/annotation/genome/neurospora/MultiHome.html).
(BZ2)

Dataset S2 High-frequency single-nucleotide polymorphisms in Louisiana strains of *N. crassa*. Data are as in Dataset S1 except that only variants at which the minor allele is present in at least 25% of strains are shown.
(BZ2)

Dataset S3 Genome-wide association analysis of germling communication among Louisiana *N. crassa* strains. Each row reports the results of a Fisher's exact test of the association between inheritance at the indicated high-frequency single-nucleotide polymorphism (SNP; see Dataset S2) and germling communication (see Table S2). For a given row, the first column reports the SNP position (see Dataset S2) and the second column reports the nominal association *p*-value.
(TXT)

Figure S1 Phylogenetic relationships between Louisiana isolates of *Neurospora crassa*. Shown is the approximate neighbor-joining genome tree of *N. crassa* Louisiana isolates inferred by FastTree [80] using all single-nucleotide polymorphisms in genic regions ascertained by RNAseq (see methods), and visualized using iTOL [81]. The branch length between a given pair of nodes is proportional to the number of segregating sites separating the individuals. Isolates indicated in red were members of the 24 strains evaluated for fusion frequency that are listed in Table S2.
(TIF)

Figure S2 Map of genic variants in 112 Louisiana isolates of *N. crassa*. Each tick mark represents the chromosomal position of one single-nucleotide polymorphism ascertained from RNAseq of wild *N. crassa* strains. Each horizontal display reports the variants along one chromosome as indicated.
(TIF)

Figure S3 Introduction of *cse-1* or *spr-7* into *Δcse-1* or *Δspr-7* mutants, respectively, restores wild type fusion frequencies. Measurements are as in Figure 5 of main text, except that the third and fifth bars represent complementation strains for *cse-1* and *spr-7*, respectively. Asterisks indicate strains with communication frequencies significantly different from that of the wild type strain from which the deletion mutants are derived [69] (Student's t-test, *p*<0.05). Bars indicate standard errors.
(TIF)

Table S1 Wild *Neurospora crassa* isolates used in this study.
(DOCX)

Table S2 Germling pair fusion frequency for 24 *Neurospora crassa* isolates from Louisiana.
(DOCX)
