# Supplementary figures and images for: Genome Wide Association Identifies Novel Loci Involved in Fungal Communication
Source: PLoS Genet. 2013 Aug 1;9(8):e1003669. doi: 10.1371/journal.pgen.1003669 (PMC3731230; doi:10.1371/journal.pgen.1003669)

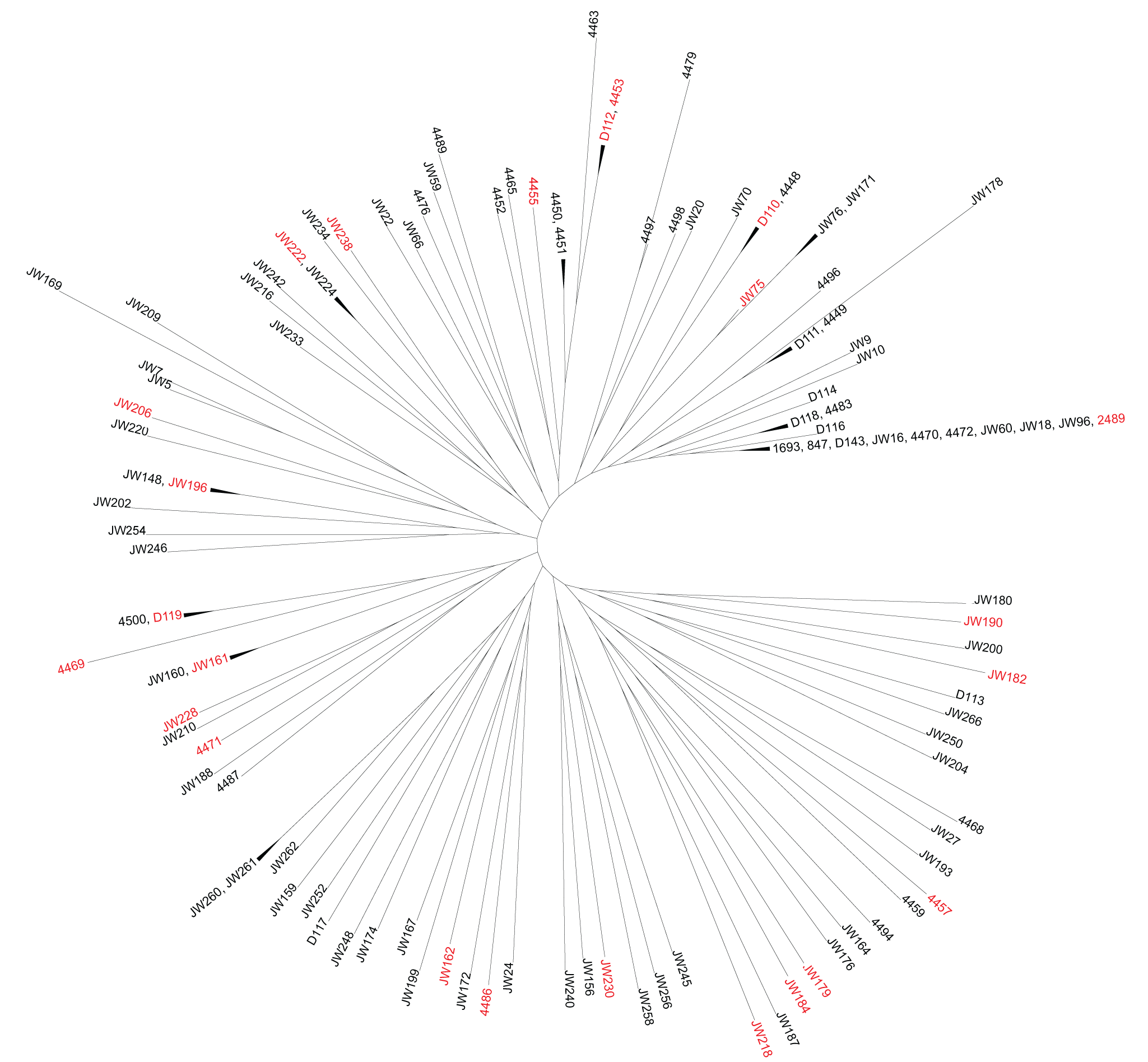

Supplement: Figure S1 — Phylogenetic relationships between Louisiana isolates of Neurospora crassa. Shown is the approximate neighbor-joining genome tree of N. crassa Louisiana isolates inferred by FastTree [80] using all single-nucleotide polymorphisms in genic regions ascertained by RNAseq (see methods), and visualized using iTOL [81]. The branch length between a given pair of nodes is proportional to the number of segregating sites separating the individuals. Isolates indicated in red were members of the 24 strains evaluated for fusion frequency that are listed in Table S2. (TIF) [file pgen.1003669.s004.tif]

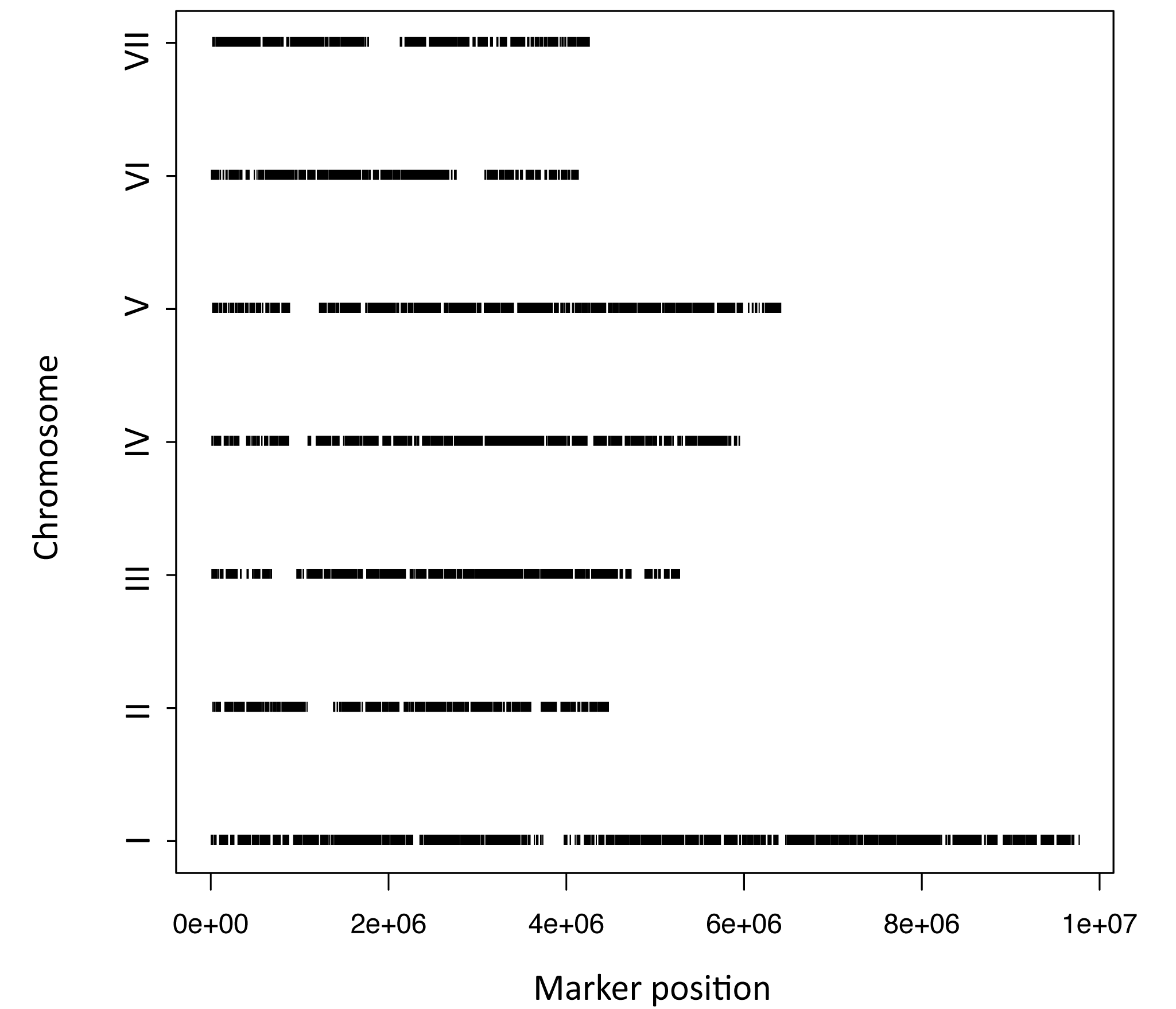

Supplement: Figure S2 — Map of genic variants in 112 Louisiana isolates of N. crassa. Each tick mark represents the chromosomal position of one single-nucleotide polymorphism ascertained from RNAseq of wild N. crassa strains. Each horizontal display reports the variants along one chromosome as indicated. (TIF) [file pgen.1003669.s005.tif]

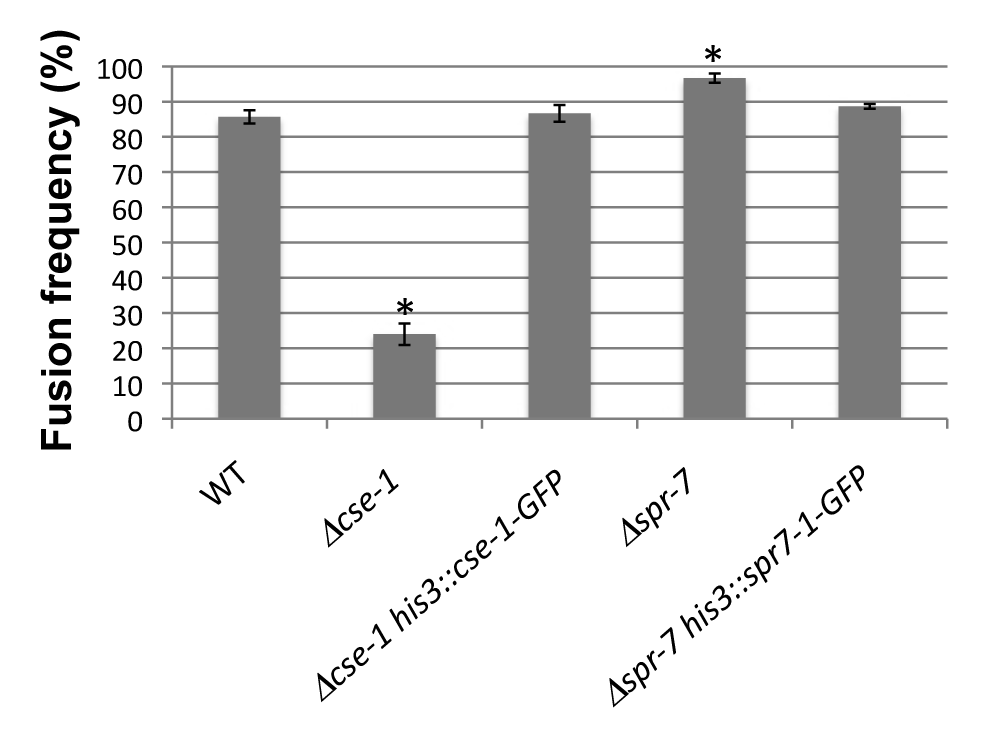

Supplement: Figure S3 — Introduction of cse-1 or spr-7 into Δcse-1 or Δspr-7 mutants, respectively, restores wild type fusion frequencies. Measurements are as in Figure 5 of main text, except that the third and fifth bars represent complementation strains for cse-1 and spr-7, respectively. Asterisks indicate strains with communication frequencies significantly different from that of the wild type strain from which the deletion mutants are derived [69] (Student's t-test, p<0.05). Bars indicate standard errors. (TIF) [file pgen.1003669.s006.tif]
