# Supplementary material for: Genome Wide Association Identifies Novel Loci Involved in Fungal Communication
Source: PLoS Genet. 2013 Aug 1;9(8):e1003669. doi: 10.1371/journal.pgen.1003669 (PMC3731230; doi:10.1371/journal.pgen.1003669)
Supplement: Table S1 — Wild Neurospora crassa isolates used in this study. (DOCX) [file pgen.1003669.s007.docx]

**Table S1. Wild *Neurospora crassa* isolates used in this study.**

| **Strain** | **Collection Site** | **Strain** | **Collection Site** |
| --- | --- | --- | --- |
| D*110 | Franklin, LA | JW 209 | Fred, LA |
| D 111 | Franklin, LA | JW 210 | Franklin, LA |
| D 112 | Franklin, LA | JW 216 | Welsh, LA |
| D 113 | Franklin, LA | JW 218 | Welsh, LA |
| D 114 | Franklin, LA | JW 220 | Roanoke, LA |
| D 116 | Franklin, LA | JW 222 | Coon, LA |
| D 117 | Franklin, LA | JW 224 | Coon, LA |
| D 118 | Franklin, LA | JW 228 | Georgia Plantation, LA |
| D 119 | Franklin, LA | JW 230 | Georgia Plantation, LA |
| D 143 | Marrero, LA | JW 233 | Houma, LA |
| JW^#^05 | Panama | JW 234 | Houma, LA |
| JW 07 | Panama | JW 238 | Welsh, LA |
| JW 09 | Welsh, LA | JW 240 | Roanoke, LA |
| JW 10 | Welsh, LA | JW 242 | Welsh, LA |
| JW 16 | Welsh, LA | JW 245 | Welsh, LA |
| JW 18 | Sugartown, LA | JW 246 | Welsh, LA |
| JW 20 | Ravenswood, LA | JW 248 | Roanoke, LA |
| JW 22 | Elizabeth, LA | JW 250 | Sugartown, LA |
| JW 24 | Welsh, LA | JW 252 | Sugartown, LA |
| JW 27 | Bayou Chicot, LA | JW 254 | Iowa, LA |
| JW 59 | Coon, LA | JW 256 | Iowa, LA |
| JW 60 | Coon, LA | JW 258 | Iowa, LA |
| JW 66 | Sugartown, LA | JW 260 | Elizabeth, LA |
| JW 70 | Coon, LA | JW 261 | Elizabeth, LA |
| JW 75 | Houma, LA | JW 262 | Elizabeth, LA |
| JW 76 | Houma, LA | JW 266 | Georgia Plantation, LA |
| JW 96 | Roanoke, LA | FGSC^+^ 847 | LA |
| JW 148 | Northside Planting, LA | FGSC 1693 | LA |
| JW 156 | Houma, LA | FGSC 2489 | Laboratory strain |
| JW 159 | Houma, LA | FGSC 4448 | Franklin, LA |
| JW 160 | Iowa, LA | FGSC 4449 | Franklin, LA |
| JW 161 | Iowa, LA | FGSC 4450 | Franklin, LA |
| JW 162 | Iowa, LA | FGSC 4451 | Franklin, LA |
| JW 164 | Marrero, LA | FGSC 4452 | Franklin, LA |
| JW 167 | Roanoke, LA | FGSC 4453 | Franklin, LA |
| JW 168 | Houma, LA | FGSC 4455 | Franklin, LA |
| JW 169 | Houma, LA | FGSC 4457 | Franklin, LA |
| JW 171 | Houma, LA | FGSC 4459 | Franklin, LA |
| JW 172 | Houma, LA | FGSC 4463 | Franklin, LA |
| JW 174 | Houma, LA | FGSC 4465 | Franklin, LA |
| JW 176 | Welsh, LA | FGSC 4468 | Franklin, LA |
| JW 178 | Roanoke, LA | FGSC 4469 | Franklin, LA |
| JW 179 | Roanoke, LA | FGSC 4470 | Franklin, LA |
| JW 180 | Roanoke, LA | FGSC 4471 | Franklin, LA |
| JW 182 | Iowa, LA | FGSC 4472 | Franklin, LA |
| JW 184 | Iowa, LA | FGSC 4476 | Franklin, LA |
| JW 187 | Elizabeth, LA | FGSC 4479 | Franklin, LA |
| JW 188 | Elizabeth, LA | FGSC 4483 | Franklin, LA |
| JW 190 | Elizabeth, LA | FGSC 4486 | Franklin, LA |
| JW 193 | Elizabeth, LA | FGSC 4487 | Franklin, LA |
| JW 196 | Northside Plantation, LA | FGSC 4489 | Franklin, LA |
| JW 199 | Franklin, LA | FGSC 4494 | Franklin, LA |
| JW 200 | Houma, LA | FGSC 4496 | Franklin, LA |
| JW 202 | Elizabeth, LA | FGSC 4497 | Franklin, LA |
| JW 204 | Bayou Chicot, LA | FGSC 4498 | Franklin, LA |
| JW 206 | Coon, LA | FGSC 4500 | Franklin, LA |

* Strains described in Dettman et al., [19].

^#^ Single conidial isolates derived from Louisiana strains [19].

^+^ Fungal Genetics Stock Center strain numbers (http://www.fgsc.net)
