# Supplementary material for: Genome Wide Association Identifies Novel Loci Involved in Fungal Communication
Source: PLoS Genet. 2013 Aug 1;9(8):e1003669. doi: 10.1371/journal.pgen.1003669 (PMC3731230; doi:10.1371/journal.pgen.1003669)
Supplement: Table S2 — Germling pair fusion frequency for 24 Neurospora crassa isolates from Louisiana. (DOCX) [file pgen.1003669.s008.docx]

**Table S2. Germling pair fusion frequency for *Neurospora crassa* isolates**

| **Strain** | **Fusion frequency (%) ± SE** | **Origin** |
| --- | --- | --- |
| FGSC^+^ 2489 | 89 ± 1 | Laboratory strain (originally from LA) |
| D* 110 | 89± 1 | Franklin, LA |
| D 112 | 60± 6 | Franklin, LA |
| D 119 | 86 ± 2 | Franklin, LA |
| FGSC 4455 | 52 ± 8 | Franklin, LA |
| FGSC 4457 | 89± 1 | Franklin, LA |
| FGSC 4453 | 59 ± 5 | Franklin, LA |
| FGSC 4469 | 80 ± 0 | Franklin, LA |
| FGSC 4471 | 76 ± 2 | Franklin, LA |
| FGSC 4486 | 91± 1 | Franklin, LA |
| JW^#^ 75 | 54 ± 4 | Houma, LA |
| JW 161 | 89± 3 | Iowa, LA |
| JW 162 | 83 ± 1 | Iowa, LA |
| JW 179 | 83 ± 6 | Roanoke, LA |
| JW 182 | 57 ± 5 | Iowa, LA |
| JW 184 | 83 ± 11 | Iowa, LA |
| JW 190 | 81 ± 10 | Elizabeth, LA |
| JW 196 | 49 ± 1 | Northside Planting, LA |
| JW 206 | 69 ± 3 | Coon, LA |
| JW 218 | 76 ± 14 | Welsh, LA |
| JW 222 | 50 ± 0 | Coon, LA |
| JW 228 | 50 ± 8 | Georgia Plantation, LA |
| JW 230 | 79 ± 3 | Georgia Plantation, LA |
| JW 238 | 38 ± 4 | Welsh, LA |

* Strains described in Dettman et al., [19].

^#^ Single conidial isolates derived from Louisiana strains [19].

^+^ Fungal Genetics Stock Center strain numbers (http://www.fgsc.net)
